# Effect of Diabetes/Hyperglycemia on the Rat Retinal Adenosinergic System

**DOI:** 10.1371/journal.pone.0067499

**Published:** 2013-06-28

**Authors:** Joana Vindeirinho, Gabriel N. Costa, Mariana B. Correia, Cláudia Cavadas, Paulo F. Santos

**Affiliations:** 1 CNC – Center for Neuroscience and Cell Biology, University of Coimbra, Coimbra, Portugal; 2 Institute for Interdisciplinary Research, University of Coimbra, Coimbra, Portugal; 3 Faculty of Pharmacy, University of Coimbra, Coimbra, Portugal; 4 Department of Life Sciences, Faculty of Sciences and Technology, University of Coimbra, Coimbra, Portugal; Hanson Institute, Australia

## Abstract

The early stages of diabetic retinopathy (DR) are characterized by alterations similar to neurodegenerative and inflammatory conditions such as increased neural apoptosis, microglial cell activation and amplified production of pro-inflammatory cytokines. Adenosine regulates several physiological functions by stimulating four subtypes of receptors, A_1_AR, A_2A_AR, A_2B_AR, and A_3_AR. Although the adenosinergic signaling system is affected by diabetes in several tissues, it is unknown whether diabetic conditions in the retina can also affect it. Adenosine delivers potent suppressive effects on virtually all cells of the immune system, but its potential role in the context of DR has yet to be studied in full. In this study, we used primary mixed cultures of rat retinal cells exposed to high glucose conditions, to mimic hyperglycemia, and a streptozotocin rat model of type 1 diabetes to determine the effect diabetes/hyperglycemia have on the expression and protein levels of adenosine receptors and of the enzymes adenosine deaminase and adenosine kinase. We found elevated mRNA and protein levels of A_1_AR and A_2A_AR, in retinal cell cultures under high glucose conditions and a transient increase in the levels of the same receptors in diabetic retinas. Adenosine deaminase and adenosine kinase expression and protein levels showed a significant decrease in diabetic retinas 30 days after diabetes induction. An enzymatic assay performed in retinal cell cultures revealed a marked decrease in the activity of adenosine deaminase under high glucose conditions. We also found an increase in extracellular adenosine levels accompanied by a decrease in intracellular levels when retinal cells were subjected to high glucose conditions. In conclusion, this study shows that several components of the retinal adenosinergic system are affected by diabetes and high glucose conditions, and the modulation observed may uncover a possible mechanism for the alleviation of the inflammatory and excitotoxic conditions observed in diabetic retinas.

## Introduction

Diabetic retinopathy (DR) is one of the major and most serious complication of both type 1 and type 2 diabetes. After 20 years of diabetes, nearly all patients with type 1 and more than 60% of patients with type 2 diabetes have some degree of retinopathy, the most frequent cause of new cases of blindness among adults aged 20–74 years. Recently, it has become apparent that cells of the neuroretina are affected in diabetes, causing subtle impairments in vision preceding the more detectable vascular lesions, an alteration that seems to happen before the blood-retinal barrier is significantly affected [1], [2]. In fact, there are several degenerative changes occurring early on, usually associated with neurodegenerative and inflammatory conditions, such as deregulation of glutamate metabolism and signaling, increased neural apoptosis, microglial cell activation and amplified production of pro-inflammatory cytokines, such as tumor necrosis factor-α (TNF-α) [3]–[7].

Adenosine, a purine nucleoside, regulates a variety of physiological functions by stimulating specific extracellular receptors. Under adverse conditions such as inflammation, adenosine production by damaged neurons is increased and helps to protect tissue against excessive damage [8]. Adenosine delivers potent suppressive effects on virtually all cells of the immune system by interacting with four subtypes of receptors, A_1_AR, A_2A_AR, A_2B_AR, and A_3_AR, and retinal microglia possess all four adenosine receptors [9], [10]. Therefore, adenosine could play a protective role in DR, acting by preventing excessive cytokine release and therefore extensive cell death. Previous studies have reported compelling evidence that diabetes can modulate the density and activity of several components of the adenosinergic signaling system in different tissues [11]–[13]. However, in the retina it is unknown what effect diabetes exerts on the whole adenosinergic system and if its modulation can have protective effects. Before tackling the potential for protection, it is first necessary to investigate if diabetic, or hyperglycemia can trigger modifications in the adenosinergic system with potential pathophysiological implications for DR.

Accordingly, in this study we evaluated the effect diabetes/hyperglycemia, considered the main cause of diabetes complications, have on the expression and protein levels of adenosine receptors and of the enzymes adenosine deaminase (ADA) and adenosine kinase (AK).

## Materials and Methods

### Ethics Statement

Experiments were performed according to the European Council directive 86/609/EEC and the legislation Portaria n. 1005/92, issued by the Portuguese Government for the protection of animals used for experimental and other scientific purposes. The procedures were approved by the CNC Committee for Animal Welfare and Protection. Animal handlers and the authors PS, GNC and CC are credited by the European Federation for Laboratory Animal Research (FELASA) category C for animal experimentation (accreditation no. 020/08). Neonatal rats were sacrificed by decapitation and adult animals were sacrificed by cervical dislocation followed by decapitation.

### Materials

Fetal Bovine Serum (FBS) was obtained from Biochrom AG (Berlin, Germany). Trypsin UPS grade was obtained from GIBCO GRL (Paisley, Scotland). Adenosine was purchased from Sigma-Aldrich (St. Louis, MO, USA). Polyvinylidene difluoride (PVDF) membranes were purchased from Millipore (Madrid, Spain). Enhanced chemifluorecense substrate (ECF) was purchased from GE Healthcare (Hertfordshire, UK). Triton X-100 and fatty acid-free bovine serum albumin (BSA) were purchased from Merck (Darmstadt, Germany). The following antibodies were used: rabbit anti-A_1_AR (1∶500; Alomone Labs, Jerusalem, Israel or 1∶1000; Calbiochem, San Diego, CA, USA), rabbit anti-A_2A_AR (1∶500; Santa Cruz Biotechnology, Santa Cruz, CA, USA), rabbit anti-A_2B_AR (1∶1000; Alomone Labs), rabbit anti-A_3_AR (1∶500; Alomone Labs), rabbit anti-ADA (1∶500; Santa Cruz), goat anti-AK (1∶400; Santa Cruz), mouse anti-actin (1∶20000; Invitrogen, Carlsbad, CA, USA). The secondary antibodies were the alkalyne phosphatase-conjugated: goat anti-rabbit (1∶20000) and rabbit anti-mouse (1∶20000), both from GE Healthcare, and rabbit anti-goat (1∶5000) from Santa Cruz.

All other reagents were obtained from Fisher Scientific, Sigma-Aldrich and Merck.

### Retinal Cell Culture

Cell cultures were obtained from 3–5 days old newborn Wistar rats, as previously described [14]. Briefly, neonatal rats were sacrificed by decapitation, the eyes were removed and the retinas dissected in Ca^2+^- and Mg^2+^-free Hank’s balanced salt solution (in mM: 137 NaCl, 5.4 KCl, 0.45 KH_2_PO_4_, 0.34 Na_2_HPO_4_, 4 NaHCO_3_, 5 glucose; pH 7.4) under sterile conditions, using a light microscope, followed by digestion with 0.05% trypsin (w/v) for 15 min at 37°C. After isolation, the cells were plated at a density of 2.0×10^6^ cells/cm^2^ on plastic multi-well plates coated with poly-D-lysine (0.1 mg/ml) and were cultured in Eagle’s minimum essential medium, supplemented with 10% (v/v) fetal bovine serum (FBS), 100 U/ml penicillin, 100 µg/ml streptomycin, 25 mM HEPES and 26 mM NaHCO_3_. Cells were then maintained at 37°C, in a humidified atmosphere of 95% air and 5% CO_2_. After two days, the culture medium was supplemented with 25 mM D-glucose (reaching a final concentration of 30 mM), to simulate high glucose conditions observed in diabetes, or with 25 mM D-mannitol, used as an osmotic control. The concentration of glucose in control and mannitol conditions was 5 mM. The cells were used for experimentation seven days after these incubations (nine days after isolation).

### Experimental Animals

Eight week old male Wistar rats, purchased from Charles River Laboratories (Spain), were handled in accordance with the European Council directive 86/609/EEC. Animals had free access to water and food in an air-conditioned room on a 12-h light–dark cycle. Diabetes mellitus was induced with a single intravenous injection of streptozotocin (65 mg/kg body weight diluted in sodium citrate 10 mM, pH 4.5). Weight and blood glucose levels were assessed for each animal on the day of injection and two days afterwards to confirm the effects of the drug. Animals were considered diabetic when presenting blood glucose levels above 250 mg/dL. The animals were maintained on a regular chow diet, *ad libitum,* for 7 or 30 days, after which the animals were sacrificed and the retinas were removed.

### Western Blot

Cells were lysed using a lysis buffer containing 137 mM NaCl, 20 mM Tris, 1% Nonidet P-40 (v/v), 10% glycerol, supplemented with protease inhibitors. Protein concentration was determined by the BCA method. Samples were separated by SDS-PAGE (10% or 7,5% according to the targeted proteins) and electroblotted to PVDF membranes (Millipore, Bedford, MA, USA). The membranes were blocked for at least 1 h at room temperature in Tris buffered saline with 0.1% Tween-20 (TBS-T) containing 5% low-fat dry milk and then incubated overnight with TBS-T supplemented with 1% low-fat dry milk containing the primary Ab. After three washes of 15 min each in TBS-T, the membranes were incubated with peroxidase-conjugated anti-rabbit or anti-mouse IgG Abs, respectively, for 1 h. After rinsing in three times TBS-T for 15 min, protein immunoreactive bands were visualized using the ECF system (GE Healthcare), on a gel imager (Versa Doc Imaging System; Bio-Rad), and digital quantification was performed using Quantity One software (Bio-Rad).

### Real Time-PCR

Total RNA was isolated from retinal cells cultured in 35 mm well plates, using the RNeasy Mini Kit from Qiagen according to the manufacturer’s instructions. Isolated RNA was eluted in RNase-free water and its quality and integrity was assessed by agarose gel electrophoresis. Total RNA was prepared from retinas of diabetic and control animals with Trizol reagent (1 ml; Sigma-Aldrich) according to the instructions of the manufacturer. The quality of the RNA was analyzed by agarose gel electrophoresis. The A260/A280 ratio of optical density was measured using the NanoDrop (Thermo-Scientific, Wilmington, DE, USA) and was between 1.9 and 2.1 for all RNA samples, indicating sufficient quality. For cDNA synthesis, 1 µg of RNA was used and reverse transcription carried out using the iScript-cDNA synthesis (Bio-Rad). Synthesized cDNA, diluted to 1∶10, was used for the amplification of the desired genes using a 3 step protocol, consisting of a 10 s denaturation step at 95°C, followed by 30 s at the annealing temperature optimal for each primer and lastly a 30 s step at 72°C for elongation. This protocol was performed with the IQ5 Multi-Color Real-Time PCR Detection System (BioRad). The PCR reaction-mix contained 2 µl of cDNA, specific primer set (2.5 µM each), 4 µl of RNase-free water and 10 µl of SYBR Green PCR Kit (BioRad) in a final volume of 20 µl. The primers used were (5′–3′): A_1_AR – (fw) GATACCTCCGAGTCAAGATCC (rv) AAACATGGGTGTCAGGCC; A_2A_AR – (fw) TCTTCGCCTGTTTTGTCCTG (rv) ACCTGTCACCAAGCCATTG; A_2B_AR – (fw) GCTCCATCTTTAGCCTCTTGG (rv) TCTTGCTCGTGTTCCAGTG; A_3_AR – (fw) TCTTCACCCATGCTTCCATC (rv) CAGAAAGGACACTAGCCAGC; ADA – (fw) GAATCCCAAAACGACGCATG (rv) CACGGTGGACTTGAAGATGAG; AK – (fw) AGAAAGACGCAGGCTCTTC (rv) AAGACAGGGAAAGCAGTGAC. The experiments were carried out in triplicate. The amplified samples were analyzed by melting-curve and a standard agarose gel electrophoresis.

Data from the target genes were normalized using the expression of three stable reference genes (TATA box binding protein, TBP; Peptidylprolyl isomerase A, Ppia; hypoxanthine guanine phosphoribosyl transferase 1, Hprt1) and the mRNA level ratios calculated using the altered Pfaffl model described previously [15], for normalizations with multiple reference genes.

### Adenosine Deaminase Activity

Cell lysates were prepared in 50 mM Tris-HC, supplemented with protease inhibitors, pH 7.2 and cleared of their insoluble fraction by centrifugation (3000 g, 10 min, 4°C). The protein concentration was determined by BCA method using the BCA protein kit and its recommended protocol. The protein content in the samples used was between 0.7 and 0.9 mg/ml. The activity of ADA was determined according to Giusti and Galanti [16], based on the Bertholet reaction. Briefly, 100 µl of the samples were added to 500 µl of a solution of 21 mM of adenosine in 50 mM phosphate buffer [NaH_2_PO_4_(H_2_O) 4.73 g/L, Na_2_HPO_4_(H_2_O)_12_ 5.62 g/L], pH 6.5 and were incubated at 37°C for 60 min. For a standard, a solution of ammonium sulphate [(NH_4_)_2_SO_4_ 75 mM in phosphate buffer] was used and for a reagent control, phosphate buffer was used. No samples were added to these two conditions nor to the sample control, which contained only adenosine solution. Afterwards, 1.5 ml of a phenol solution (106 mM phenol, 170 µM sodium nitroprusside) and a sodium hypochlorite solution (11 mM NaOCl, 125 mM NaOH) were added and the samples incubated at 37°C for 30 min. The final products were quantified spectrophotometrically at a wavelength of 620 nm. Results were calculated according to the formula: [(sample–sample control)/(standard–reagent control)] ×50; results were expressed in percentage of control [11].

### Adenosine Quantification

Cells were lysated with 0.6 M perchloric acid, supplemented with 25 mM EDTA-Na^+^, and centrifuged at 14,000 g for 2 min at 4°C, according to previously described methods [13]. The resulting pellet was solubilized with 1 M NaOH for total protein analysis by the BCA method. After neutralization of the supernatant with 3 M KOH in 1.5 M Tris, the samples were centrifuged at 14000 g for 2 min (0–4°C). To determine extracellular accumulation of adenosine, medium was recovered prior to the above procedure. The resulting supernatants and medium samples were assayed for adenosine concentration by separation in a reverse-phase high-performance liquid chromatography (HPLC), with detection at 254 nm. The HPLC apparatus was a Beckman-System Gold with a computer controlled 126 Binary Pump Model and 166 Variable UV detector. The column used was a Lichrospher 100 RP-18 (5 µm) from Merck. An isocratic elution with 10 mM phosphate buffer (NaH_2_PO_4_; pH 6.0) and 14% methanol was performed with a flow rate of 1.5 ml/min, and each analysis took 5 minutes. Adenosine was identified by retention time, absorption spectra and correlation with standards.

## Results

We have previously shown that diabetes or elevated glucose levels alters the retinal purinergic system, namely, the extracellular levels of ATP [17] and the content of P2 receptors [18], but the effect on the P1 receptors has not been addressed yet, despite the known influence that both branches of the purinergic system exercise on each other. Therefore, we evaluated the effect of diabetes or high glucose exposure on the retinal adenosine A_1_AR, A_2A_AR, A_2B_AR, A_3_AR protein and mRNA levels by Western blot and quantitative RT-PCR analysis.

### Effect of High Glucose on the Protein and mRNA Levels of Adenosine Receptors A_1_AR, A_2A_AR, A_2B_AR or A_3_AR in Retinal Cell Cultures

**Figure 1:**
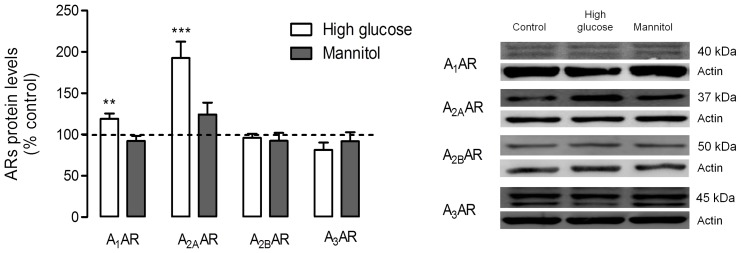
Adenosine receptor protein levels in cultured retinal cells. Cells were incubated with 30 mM glucose to mimic hyperglycemic conditions for a period of 7 days. Osmotic control was performed by incubating cells with 25 mM mannitol. 60 µg for A_1_AR and 50 µg for A_2A_AR, A_2B_AR and A_3_AR of protein content from each sample was electrophoresed and probed for the presence of the respective receptor. Total protein levels were normalized by the loading control (actin), and expressed as percentage of the control group. The mean±SEM of 4–7 independent experiments was analyzed with one-way ANOVA test and Tukey’s multiple comparison test. **p<0.01, ***p<0.001.

As illustrated in Fig. 1, the exposure of retinal cell cultures to high glucose levels (30 mM), used to mimic the hyperglycemic conditions observed in diabetes, induced an increase in the protein levels of A_1_AR and A_2A_AR adenosine receptors, but did not significantly alter the content of A_2B_AR or A_3_AR. The protein levels of A_1_AR were increased up to 119.1±6.4% of control (*p<0.01*), while the A_2A_AR protein levels almost doubled, reaching 192.5±19.9% of control (*p<0.001*) in cells cultured in high glucose conditions. These results were not due to an osmotic effect, since retinal cells cultured in medium containing 25 mM mannitol, used as an osmotic control, did not show any variation from control levels.

**Figure 2:**
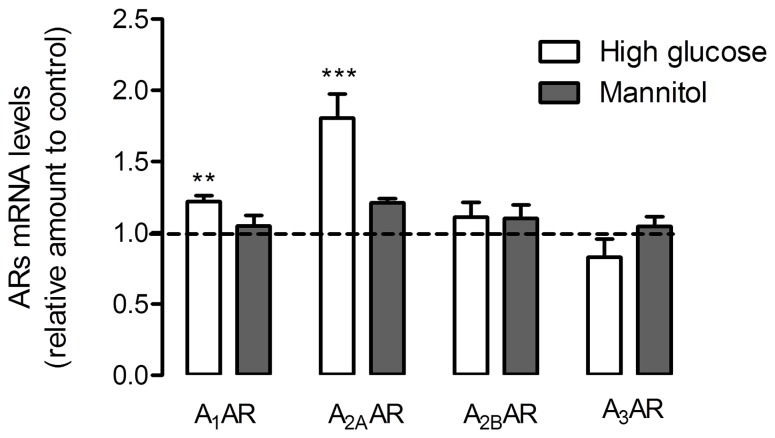
Adenosine receptor expression levels in cultured retinal cells. Cells were treated as previously described in Figure 1. Total RNA was isolated using the RNeasy Mini Kit from Qiagen according to the manufacturer’s instructions. Data from the target genes were normalized using the expression of three stable reference genes and the mRNA level ratios calculated using the altered Pfaffl model for normalizations with multiple reference genes. Experiments were carried out in triplicate. The mean±SEM of 3–4 independent experiments was analyzed with one-way ANOVA test and Tukey’s multiple comparison test. **p*<*0.01, ***p<0.001.

The analysis of the expression levels of all four adenosine receptors using quantitative RT-PCR in retinal cell cultures showed results similar to those observed for the receptors’ protein levels described above. As illustrated in Fig. 2, in cell cultures subjected to high glucose conditions, both A_1_AR and A_2A_AR expression levels increased (1.22±0.04 fold of control (*p<0.01*) for A_1_AR, and 1.81±0.17 (*p<0.001*) for A_2A_AR). On the other hand, A_2B_AR and A_3_AR expression levels were not altered in high glucose conditions. Similar to what was observed for the receptors protein levels, the alterations occurring to A_1_AR and A_2A_AR expression levels were not due to changes in osmolarity since cells incubated with mannitol did not present expression levels different from those observed in cells cultured in control conditions.

### Effect of Diabetes on the Protein and mRNA Levels of Adenosine Receptors in Diabetic Retinas

**Figure 3:**
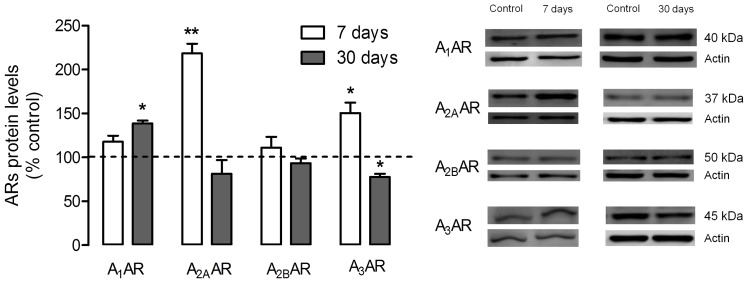
Effect of streptozotocin-induced diabetes on retinal adenosine receptor protein levels. Rats were subjected to an intraperitoneal injection of streptozotocin (STZ) and maintained for a period of 7 days and 30 days. Protein content (50 µg) from each sample was loaded into a 7.5% gel, electrophoresed and probed for the presence of the respective receptor. Total protein levels were normalized by the loading control (actin), and expressed as percentage of the control group. The mean±SEM of 3–5 samples for each condition was analyzed with the Student’s t-test (diabetic *vs* control) and F test. *p*<*0.05 **p*<*0.01.

In whole retina extracts obtained from diabetic animals, seven days after induction of diabetes, the retinal protein levels of A_1_AR were augmented when compared to the retinas isolated from control animals (Fig. 3). However, this increment only attained statistical significance after 30 days of diabetes, [126.7±12.2% of control (*p<0.05*)]. As for the protein levels of A_2A_AR, the 2-fold increase observed in cell cultures was mirrored in the results from STZ treated rats maintained for 7 days [218.4±11.1% of control (*p<0.01*)], while after 30 days A_2A_AR in diabetic rat retinas returned to control levels. The protein levels of A_2B_AR obtained in the diabetic animals for both periods of diabetes, 7 and 30 days, were not significantly different from control conditions. The results obtained for A_3_AR in STZ treated rats maintained for a period of 7 days showed a significant increase [150.6±11.7% of control (*p<0.05*)] in A_3_AR total protein levels. Nonetheless, this effect was transient, being followed by a decrease to 77.7±3.6% of control (*p<0.05*) 30 days after diabetes induction.

**Figure 4:**
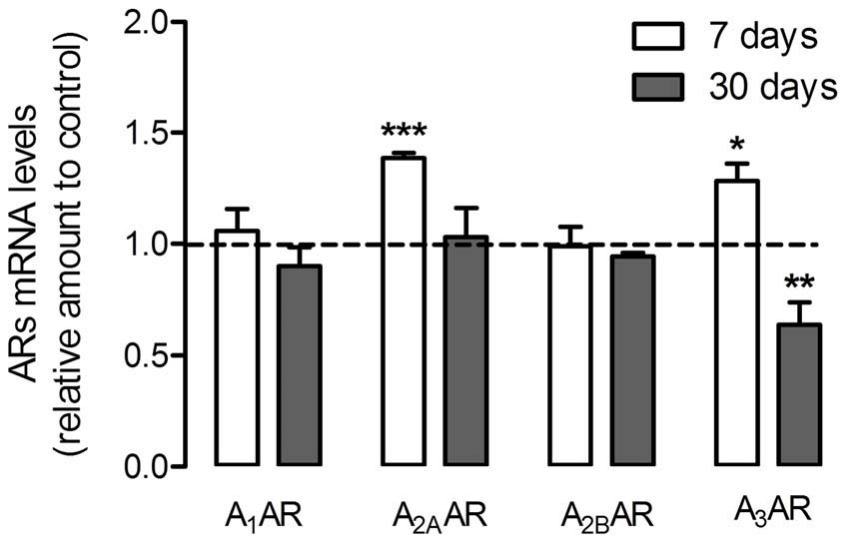
Effect of streptozotocin-induced diabetes on retinal adenosine receptor expression levels. Rats were subjected to an intraperitoneal injection of STZ and maintained for a period of 7 days and 30 days. Total RNA was isolated using Trizol reagent according to the manufacturer’s instructions. Data from the target genes were normalized using the expression of three stable reference genes and the mRNA level ratios calculated using the altered Pfaffl model for normalizations with multiple reference genes. Experiments were carried out in triplicate. The mean±SEM of 3–4 independent experiments was analyzed with the Student’s t-test (diabetic *vs* control) and F test. *p*<*0.05, **p*<*0.01, ***p*<*0.001.

The levels of the adenosine receptors, measured by quantitative RT-PCR in whole retina extracts, revealed similar alterations, with the exception of A_1_AR. As seen in Fig. 4, the A_1_AR mRNA levels were not significantly altered at 7 or 30 days. A_2B_AR expression levels also remained unchanged from 7 to 30 days after diabetes, while A_2A_AR expression levels showed an increase in the first 7 days [1.39±0.02 fold of control (*p<0.001*)], matching the increase observed in the protein levels, and expression levels similar to control levels for the longest experimental time. The expression levels of A_3_AR also corresponded to the alterations observed in the protein levels, exhibiting a transient increase at 7 days [1.28±0.08 fold of control (*p<0.05*)] followed by a significant decrease at 30 days [0.64±0.10 fold of control (*p<0.01*)].

### Effect of Diabetes/High Glucose on Adenosine Deaminase and Adenosine Kinase

Adenosine in the cell can be either phosphorylated to AMP by AK or deaminated to inosine by ADA. AK is the main pathway of adenosine metabolism in normal physiological conditions, while deamination by ADA gains a more important role when adenosine concentrations rise, such as in ischemic situations. ADA is also present in extracellular form, thereby contributing to the regulation of extracellular levels of adenosine [19], [20].

**Figure 5:**
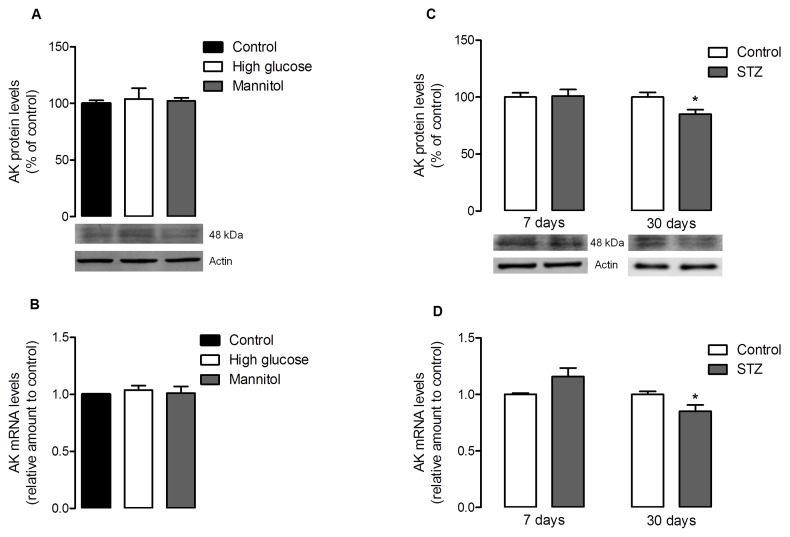
Adenosine kinase levels in cultured retinal cells and diabetic retinas. (A–B) Cells were treated as previously described in Figure 1. The mean±SEM of 4–6 independent experiments was analyzed with one-way ANOVA and Tukey’s multiple comparison test. (C–D) Rats were subjected to an intraperitoneal injection of STZ and maintained for a period of 7 days and 30 days. (**A**) 50 µg of protein content from each sample was loaded into a 7.5% gel, electrophoresed and probed for the presence of AK. Total protein levels were normalized by the loading control (actin), and expressed as percentage of the control group. (**B**) Total RNA was isolated using the RNeasy Mini Kit from Qiagen according to the manufacturer’s instructions. Data from the target gene was normalized using the expression of three stable reference genes and the mRNA level ratios calculated using the altered Pfaffl model for normalizations with multiple reference genes. Experiments were carried out in triplicate. (**C**) 50 µg of protein content from each sample was loaded into a 7.5% gel, electrophoresed and probed for the presence of AK. Total protein levels were normalized by the loading control (actin), and expressed as percentage of the control group. The mean±SEM of 3–6 samples for each condition was analyzed with the Student’s t-test (diabetic *vs* control) and F test. *p<0.05 (**D**) Total RNA was isolated using Trizol reagent according to the manufacturer’s instructions. Data from the target gene was normalized using the expression of three stable reference genes and the mRNA level ratios calculated using the altered Pfaffl model for normalizations with multiple reference genes. Experiments were carried out in triplicate. The mean±SEM of 4–5 independent experiments was analyzed with one-way ANOVA and Tukey’s multiple comparison test. *p*<*0.05.

**Figure 6:**
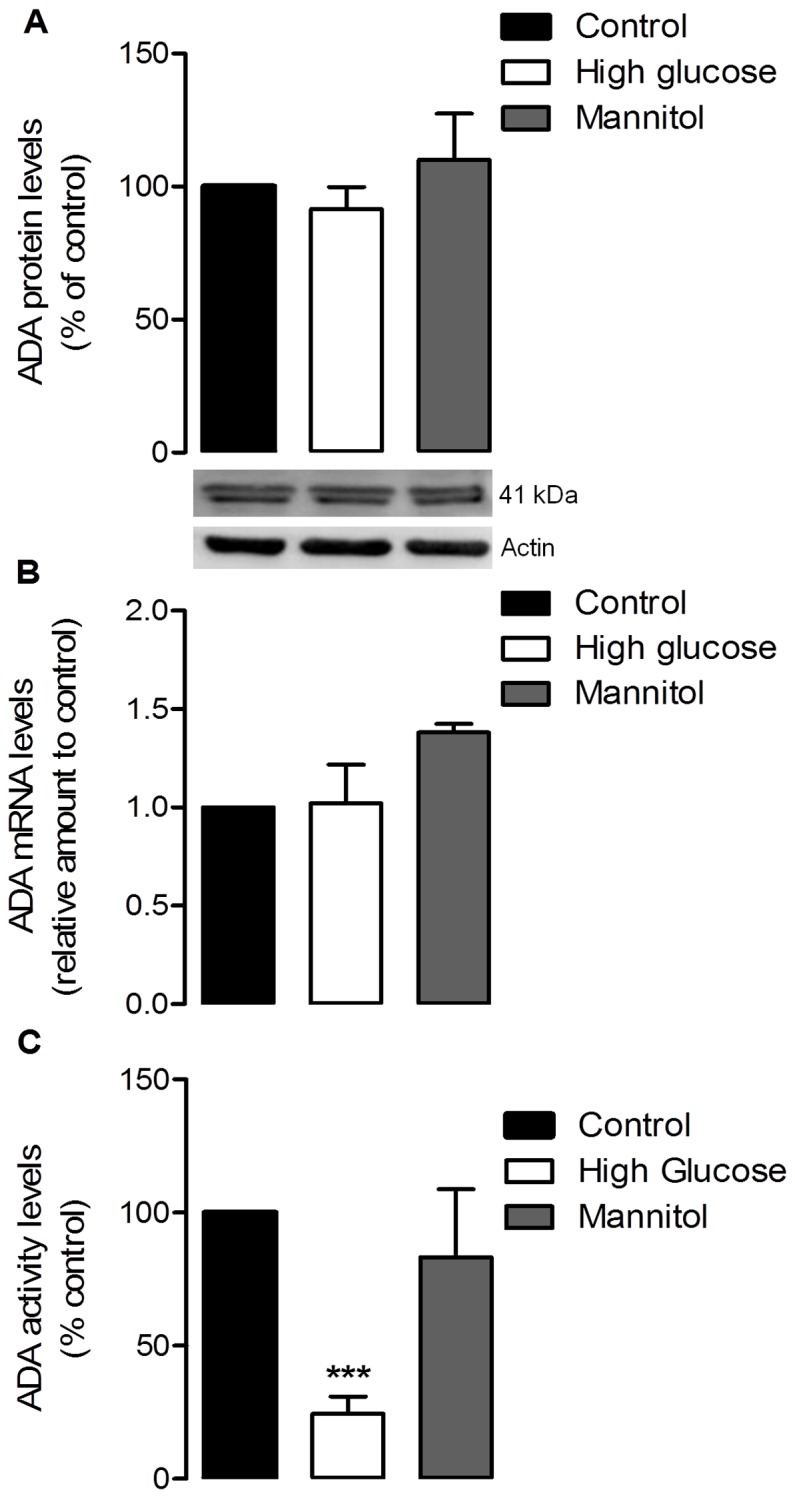
Adenosine deaminase levels in cultured retinal cells. Cells were treated as previously described in Figure 1. The mean±SEM of 4–6 independent experiments was analyzed with one-way ANOVA and Tukey’s multiple comparison test. ***p<0.001 (**A**) 50 µg of protein content from each sample was loaded into a 7.5% gel, electrophoresed and probed for the presence of ADA. Total protein levels were normalized by the loading control (actin), and expressed as percentage of the control group. (**B**) Total RNA was isolated using the RNeasy Mini Kit from Qiagen according to the manufacturer’s instructions. Data from the target gene was normalized using the expression of three stable reference genes and the mRNA level ratios calculated using the altered Pfaffl model for normalizations with multiple reference genes. Experiments were carried out in triplicate. (**C**) 100 µl of the samples were added to 500 µl of a solution of 21 mM of adenosine to determine the enzymatic activity levels of ADA based on the Bertholet reaction. The final products were quantified spectrophotometrically at a wavelength of 620 nm. Protein content in the samples was between 0.7 and 0.9 mg/ml. Results are expressed as percentage of the control group.

**Figure 7:**
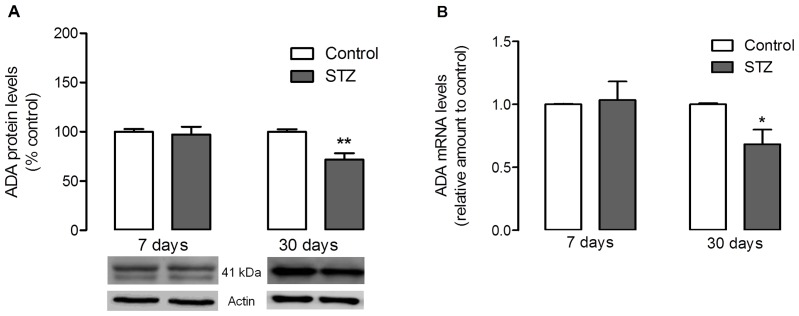
Effect of streptozotocin-induced diabetes on retinal adenosine deaminase levels. Rats were subjected to an intraperitoneal injection of STZ and maintained for a period of 7 days and 30 days. (**A**) 50 µg of protein content from each sample was loaded into a 7.5% gel, electrophoresed and probed for the presence of ADA. Total protein levels were normalized by the loading control (actin), and expressed as percentage of the control group. The mean±SEM of 4–5 samples for each condition was analyzed with the Student’s t-test (diabetic *vs* control) and F test **p<0.01 (**B**) Total RNA was isolated using Trizol reagent according to the manufacturer’s instructions. Data from the target gene was normalized using the expression of three stable reference genes and the mRNA level ratios calculated using the altered Pfaffl model for normalizations with multiple reference genes. Experiments were carried out in triplicate. The mean±SEM of 3–5 independent experiments was analyzed with one-way ANOVA and Tukey’s multiple comparison test. *p*<*0.05.

In retinal cell cultures, the protein levels of both AK and ADA were not affected by the culture conditions (Fig. 5A and 6A). The results obtained in high glucose conditions were not significantly different from control, and also similar to those observed for the osmotic control. The analysis of the expression levels of AK and ADA showed that, again, the levels of both enzymes were not affected by high glucose or osmotic control conditions in mixed retinal cell cultures (Fig. 5B and 6B). In whole retina extracts (Fig. 5C and 7A), after 7 days of diabetes induction by STZ injection, the retinal AK and ADA protein levels were not significantly different from the levels observed in control animals. However, after 30 days of diabetes induction it was observed a marked decrease in AK and ADA protein levels: AK levels decreased to 85.03±3.95% of control (*p<0.05*), and ADA levels were down to 71.8±6.4% of control (*p<0.01*). As for the expression levels of these enzymes in whole retina extracts, they also corresponded to the alterations observed in the protein levels (Fig. 5D and 7B): there were no variations at 7 days of diabetes induction, while at 30 days there was a significant decrease in the expression levels of AK [0.85±0.06 fold decrease from control (*p<0.05*)] and ADA [0.68±0.12 fold decrease from control (*p<0.05*)].

**Figure 8:**
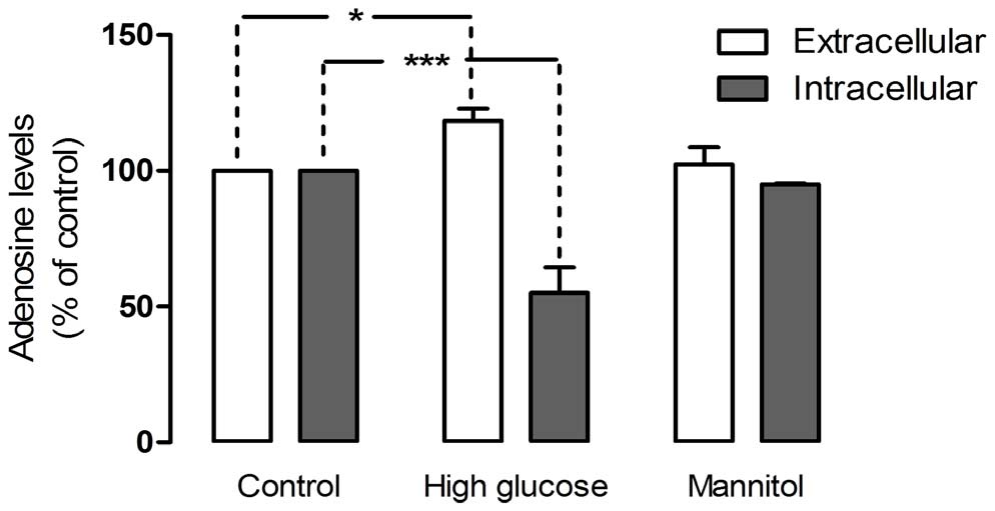
Adenosine levels in retinal cell cultures under high glucose conditions. Cells were treated as previously described in Figure 1. 20 µl of culture medium and intracellular extracts were analyzed by HPLC. Protein content in the samples was between 0.4 and 0.6 mg/ml. Results are expressed as percentage of the control group. The mean±SEM of 5–8 independent experiments was analyzed with one-way ANOVA and Tukey’s multiple comparison test. **p*<*0.01; *p<0.05.

With the protein and expression levels of ADA remaining unaltered in mixed retinal cell cultures subjected to high glucose conditions, we assessed the activity levels of ADA in those conditions, by performing the enzymatic activity assay based on the Bertholet reaction. As shown in Fig. 6C, ADA activity was severely compromised in retinal cell cultures after 7 days in high glucose conditions. Values for ADA enzymatic activity were reduced to 24.4±6.5% in relation to the control group (*p<0.001*), suggesting a decline in the adenosine removal levels during these conditions. In fact, a quantification of extracellular and intracellular adenosine levels in mixed retinal cell cultures (Fig. 8) showed that, in high glucose conditions, there is a significant increase in the levels of extracellular adenosine [118.4%±4.6% of control (*p<0.05*)], paired with a decrease in intracellular levels of this nucleoside [55.12%±9.3% of control (*p<0.001*)]. Nucleoside levels of the osmotic control in both intra and extracellular quantifications remained close to control levels.

## Discussion

The present study provides the first clear evidence that the retinal adenosinergic system is affected by diabetes. We observed that the levels of adenosine receptors are modified in rat retinal cells exposed to high glucose conditions and in the retina of type 1 diabetic rats. In particular, there was an up-regulation of the A_1_ receptors and the A_2A_receptors, indicating that both high glucose conditions and the induction of diabetes are followed by alterations that may affect the adenosine signaling mechanisms. We also observed a decrease in mRNA and protein levels of ADA and AK in retinas 30 days after diabetes induction. Furthermore, even though the levels of ADA were not significantly altered, the activity of this enzyme was severely compromised in retinal cells cultured under high glucose conditions.

Others have described a general occurrence of adaptative changes to the density of adenosine receptors upon prolonged noxious conditions [8]. Diabetes, being a prolonged condition that causes metabolic alterations to the cellular environment, is likely to influence the adenosinergic system as well. In fact, several studies show that diabetes can lead to a number of modifications of the components of this system: It was demonstrated that in the hippocampus of diabetic rats, the expression of A_1_AR was down-regulated and A_2A_AR was up-regulated [13]; Diabetic conditions can downregulate the expression and protein levels of AK in rat kidney, heart, liver, spleen and lymphocytes [21], [22]; Diabetes can also change the activities of nucleoside triphosphate diphosphohydrolases (NTPDases), ecto-nucleotide pyrophosphatase/phosphodiesterase (E-NPP), 5′-nucleotidase and ADA in platelets [11], enzymes that regulate the levels of extracellular adenosine; Also, NTPDase 1 expression and distribution levels are altered in retinal tissue from diabetic rats [12]. More recently it was shown that diabetic conditions alter the protein levels of A_2A_AR in the retina of diabetic mice [23]. The mechanism by which streptozotocin-induced diabetes and high glucose conditions lead to the observed modification of the levels of retinal adenosine receptors still remains to be established, although more than one mechanism may be involved.

The observed up-regulation of inhibitory A_1_AR in both high glucose and diabetic conditions may be related to the chronic inflammation environment created by hyperglycemic conditions in the retina. It has been shown in several reports that different agents and conditions can stimulate A_1_AR expression in an NF-κB-dependent manner [24]–[26]. NF-κB, a transcription factor with putative binding sites found in all adenosine receptor genes [27], was shown to be regulated by TNF-α in several cell types, including retinal cells [28], revealing a possible relationship between the high levels of TNF-α present in hyperglycemic conditions [4] and the increased levels of A_1_AR detected under the same conditions. Another possibility is the potential effect of the high levels of adenosine registered in retinal cultures exposed to high glucose conditions: a study performed in avian retinal cells showed a connection between high levels of A_2A_AR activation and an up-regulation of A_1_AR, via a cAMP/PKA dependent pathway [29]. This upregulation was blocked when NF-κB inhibitors were used, indicating another link between NF-κB and A_1_AR regulation.

The overall effect of A_1_AR is inhibitory, since its activation usually leads to a state of reduced activity, particularly in neurons where there is a decrease in neuronal excitability, firing rate and neurotransmitter release. The ability of this receptor to inhibit the release of retinal neurotransmitters and decrease the influx of Ca^2+^ [30] gains a new importance in these circumstances. The increase in A_1_AR levels in hyperglycemic conditions may have a neuroprotective effect, by down-regulating excessive excitatory neurotransmission and decreasing high Ca^2+^ influx levels, two features, which are consequence of diabetic conditions in the retina [29], [31], [32].

The increase in inflammatory markers induced by high glucose and diabetic conditions may also be the cause behind the elevated levels of A_2A_AR found in those conditions. A_2A_AR regulation is very sensitive to extracellular environment alterations, such as concentrations of inflammatory mediators [9] and, in a similar manner to A_1_AR, A_2A_AR, can also be regulated by the transcription factor NF-κB [33], [34]. The observed A_2A_AR levels increase, along with the A_1_AR alterations, both with a possible link to NF-κB and increased levels of TNF-α, reveals a possible negative feedback mechanism occurring when TNF-α levels are increased, with the cytokine activating the signaling pathways that work to recover the homeostatic state.

Widely regarded as a main player in inflammation, A_2A_AR signaling may have the potential to play a key role in the response to the inflammatory conditions observed in the early stages of diabetic retinopathy. A_2A_AR regulates the release of inflammatory cytokines in many immune cell types, down-regulating the levels of several pro-inflammatory cytokines, such as IL-6, IL-8, IL-12 and TNF-α [9], [10], [33], [35], [36], that are known to be increased in diabetic retinas [4]. We have previously shown that TNF-α is responsible for the rise in cell death witnessed in diabetic conditions [37]. Therefore, increasing the levels of A_2A_R may function as a protective mechanism, reducing the release of inflammatory mediators and the subsequent death of neurons observed in the diabetic retina.

Glutamate is the major excitatory neurotransmitter in the retina, being responsible for the neurotransmission from photoreceptors to bipolar cells and from bipolar cells to ganglion cells. However, increased levels of glutamate (usually resulting in excessive stimulation) are implicated in the phenomenon of excitotoxicity, which leads to neurodegeneration. There is an increase in glutamate release in retinal cultures exposed to high glucose and in retinas of diabetic animals [14], and this exposure of retinal cells to higher levels of glutamate can induce retinal cell death [38]. Adenosine can inhibit the extracellular accumulation of excitatory amino acids through the activation of A_2A_AR [39] and thus inhibit glutamate toxicity in retinal neurons [40].

Therefore, the increase of A_2A_ adenosine receptor levels may represent a mechanism for protecting retinal cells against the inflammatory effects of diabetes. Supporting this hypothesis are preliminary results where we observed that activation of A_2A_AR with a specific agonist prevented the decrease in cell viability caused by hyperglycemic conditions in retinal cells, while the blockade of A_2A_AR aggravated the loss of cell viability (data not shown).

While they were not altered in cell cultures under high glucose conditions, the protein and mRNA levels of A_3_AR were temporarily increased in the retinas of diabetic rats 7 days after the STZ injection, followed by a decrease observed 30 days after injection. This discrepancy in results may be due to humoral or neuronal signals that regulate A_3_AR levels *in vivo*, but are not present in *in vitro* conditions. Another cause may be due to the characteristics of our cell cultures: although our primary cell cultures possess all retinal cell types, their relative proportions may be different than what is found in the retina. If the increase in the A_3_AR observed in retinas is due to an effect in a particular cell type, this may not be reflected in our retinal cultures due to the differences in the proportions of cell types between cultures and retinas. The answer to this question would require a sorting of the different populations at the end point of the culture time, and it is a study planned for the near future. This would also allow us a better understanding of all the results we obtained in primary cell cultures, not just A_3_AR, to see if the alterations observed are across the board, or specific to certain cell populations.

ADA and AK are the key elements responsible for the regulation of intracellular and extracellular adenosine, by phosphorylation to AMP by AK or deamination to inosine by ADA. AK is the main pathway of adenosine metabolism in normal physiological conditions, becoming easily saturated with basal concentrations of adenosine. The role of ADA is then secondary in normal physiologic conditions, both inside and outside the cell, with the larger share of adenosine removal from the extracellular space being done by reuptake with nucleoside transporters. However, during disruptive situations, such as ischemia and hypoxia where adenosine levels rise, ADA gains a more predominant role [19], [20]. The decrease observed in the levels of both ADA and AK in diabetic retinas 30 days after STZ treatment may signal a severe dysregulation of adenosine levels in diabetic conditions, since it has been shown that a blockade of the activity of ADA and AK lead to a massive increase in adenosine concentration in various tissues [41]–[45].

Our results show that, in retinal cells, ADA activity levels plummet down in response to high glucose conditions. A low activity rate for ADA may mean a lower rate of adenosine removal from the extracellular space, which may correlate with the higher levels of extracellular adenosine concentration we observed in retinal cultures subjected to high glucose conditions, alongside with the reported increase in ATP release under the same conditions [17]. Such an increase in adenosine concentration may affect the signaling carried out by the adenosine receptors, particularly A_2B_AR and A_3_AR, the receptors with lower affinity for adenosine, possibly creating a powerful immunosuppressive response, since both receptors are also recognized as important mediators of inflammation [9], [19], [33], [46], [47]. Highlighting the decrease in activity of ADA is the fact that ADA expression and protein levels were unaltered in retinal cell cultures during high glucose conditions and in the retinas of diabetic rats at 7 days. The extracellular form of ADA does not have its own transmembrane domain, being present on the surface of many cell types by alternative anchoring mechanisms, such as association with CD26 [48] and interactions with A1AR and A2BAR [49]. The decrease observed in ADA activity under high glucose conditions can be a result of alterations to these anchoring mechanisms, or a direct influence of the anchor proteins on the activity of the enzyme. A link between the increased levels of A_1_AR observed under the same conditions and ADA activity may be one hypothesis, while the interaction between ADA and CD26 may be another: this cell surface peptidase is involved in glucose and insulin regulation mechanisms, and selective CD26 inhibitors are being studied as a safe therapy for diabetes type 2 [50], [51].

In conclusion, our work shows that the retinal adenosinergic system is affected by diabetes and high glucose conditions. The modulation observed in the expression and protein levels of the receptors A_1_AR, A_2A_AR and A_3_AR and enzymes AK and ADA, as well as the impact on ADA activity and extracellular adenosine levels, may reveal a possible mechanism for the mitigation of the inflammatory and excitotoxic conditions observed in diabetic retinas, and thus a potential tool for the prevention of the neuronal cell death that occurs in the early stages of diabetic retinopathy.
